# Pectus carinatum: When less is more

**DOI:** 10.7196/AJTCCM.2019.v25i3.019

**Published:** 2019-09-17

**Authors:** M Martinez-Ferro, G Bellia-Munzon, I A Schewitz, L Toselli

**Affiliations:** 1 Fundación Hospitalaria Mother and Child Medical Centre, Buenos Aires, Argentina; 2 Department of Cardiothoracic Surgery, School of Medicine, Faculty of Health Sciences, University of Pretoria, South Africa

**Keywords:** pectus carinatum, non-surgical

## Abstract

Awareness of pectus carinatum has increased among the medical community over the last several decades, as innovative options for nonsurgical treatments have become more widely known. Management alternatives have shifted from open resective to minimally invasive strategies, and finally, to reshaping the chest using both surgical and non-surgical modalities. We aim to review the evolution of the diagnosis and treatment of pectus carinatum up to its current management.

## Background

**Fig. 1:**
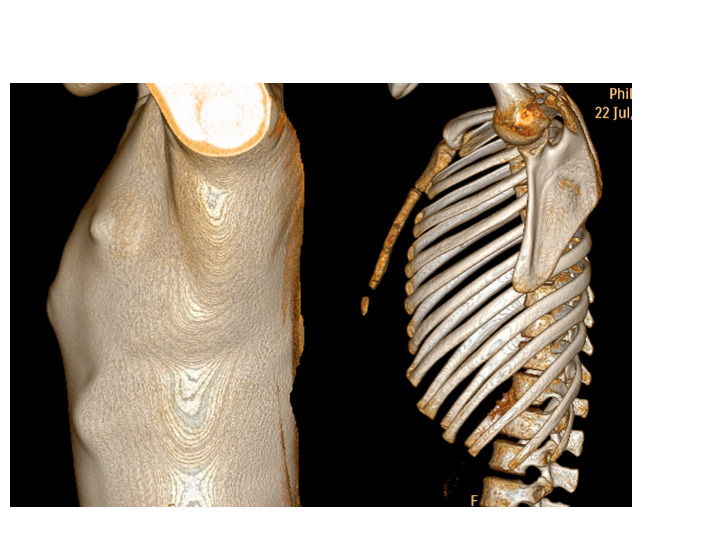
Chest computed tomography scan with 3D reconstruction of patient with pectus carinatum. This usually comprises the whole thoracic cage, including sternum and chondrocostal structures.

Although pectus carinatum has classically been seen as an isolated protrusion of the sternum, the condition comprises a deformity of the entire thoracic cage, both the sternum and the adjacent chondrocostal structures (Fig. 1). Aside from the negative aesthetic impact, patients with pectus carinatum have psychological problems, impairments in their social interactions, spinal misalignments, poor posture and back pain. While infrequent, cardiopulmonary associations have been described,^[1]^ and it is associated with syndromes such as Noonan, Marfan and von Recklinghausen, which also have a pectus deformity.^[2,3]^ In 1958, Currarino and Silverman^[4]^ described a special type of carinatum, pectus arcuatum, in association with cardiovascular anomalies. Several important developments in the diagnosis and treatment of pectus carinatum have taken place since these initial steps. Our aim is to describe these events in a way that enables paediatric and general thoracic surgeons to understand the origins and evolution of this disease and its treatment.

A Medline literature search was undertaken using the MeSH term ‘pectus carinatum’. A total of 382 articles were retrieved. A subset was chosen according to the authors’ view of the best description of the evolution of diagnosis and treatment of pectus carinatum.

## Incidence, aetiology and classification

The incidence of pectus carinatum is very difficult to estimate because the condition is often underdiagnosed. The development and spread of non-invasive treatment methods has raised interest in this malformation, and referral has increased exponentially in the last 20 years.

Pectus carinatum is considered the second-most common cause of thoracic malformations. Its reported incidence ranges from a fifth of that of pectus in some centres, to an equal incidence in others.^[5,6]^ It is more prevalent in males, at a ratio of 3 or 4 to 1.^[5,6]^

Aetiology may be primary or secondary. Primary malformations are those with no predisposing factors except for familial aggregation of 25%,^[7]^ and secondary are those malformations that occur after large sternotomies for cardiac surgeries,or resective operations for pectus excavatum repair.^[8,9]^

**Fig. 2:**
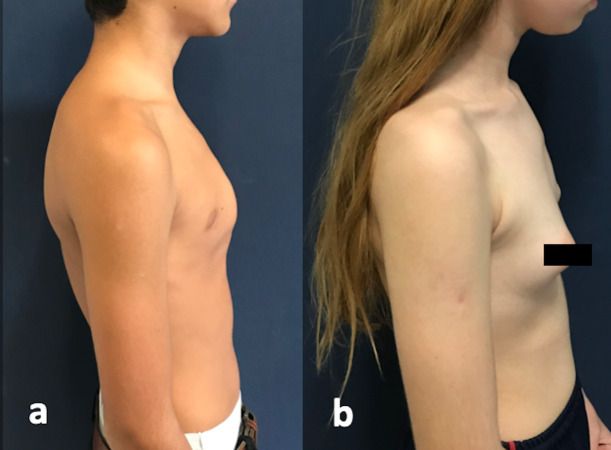
Types of pectus carinatum: (a) chondrogladiolar and (b) chondromanubrial.

Pectus carinatum may be classified as of either chondrogladiolar or chondromanubrial type, depending on the site of the sternal angulation (Fig. 2). In the chondrogladiolar type, the more common one, the acute angle of the sternum is located at the body of the sternum, while in the chondromanubrial type the angle is located cephalad, at the manubrium. The malformation may be also classified according to symmetry: symmetric; asymmetric with sternal rotation to the left or right; and mixed excavatum/carinatum.

**Fig. 3:**
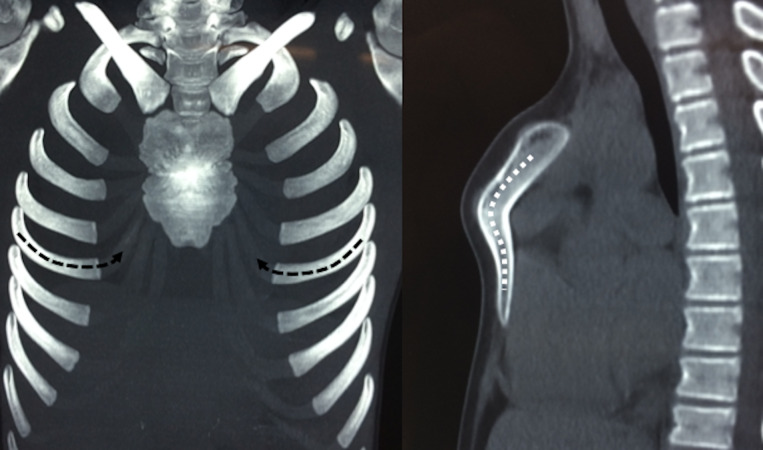
3D chest wall computed tomography scan of a patient with pectus arcuatum. This condition causes extensive deformity of adjacent chondrocostal structures. Note the deformed costal arches pointing upwards in order to connect with the shortened sternum (left; black dotted lines). This characteristic short, wide and non-segmented sternum is usually severely bent (right; white dotted lines).

Special malformations include pectus arcuatum (Fig. 3), which consists of a short, thick and wide non-segmented sternum with marked posterior angulation at the site of the normal chondromanubrial junction. When associated with cardiac anomalies, this is referred to as Currarino-Silverman syndrome.^[4]^

Diagnosis is made by physical examination; the deformity can be characterised without complementary studies such as X-ray or computed tomography, which can add no information and in addition require radiation. Systematic photography is used to assess the shape of the chest for the duration of the follow-up period. The elasticity of the sternum and cartilages should always be assessed; this can be achieved subjectively by manual compression or by means of pressure-measuring devices.

Only in special cases should further studies be done, such as in associated skeletal deformities, or when associated syndromes such as von Recklinghausen, Poland or Marfan, or cardiac conditions, are suspected.

**Fig. 4:**
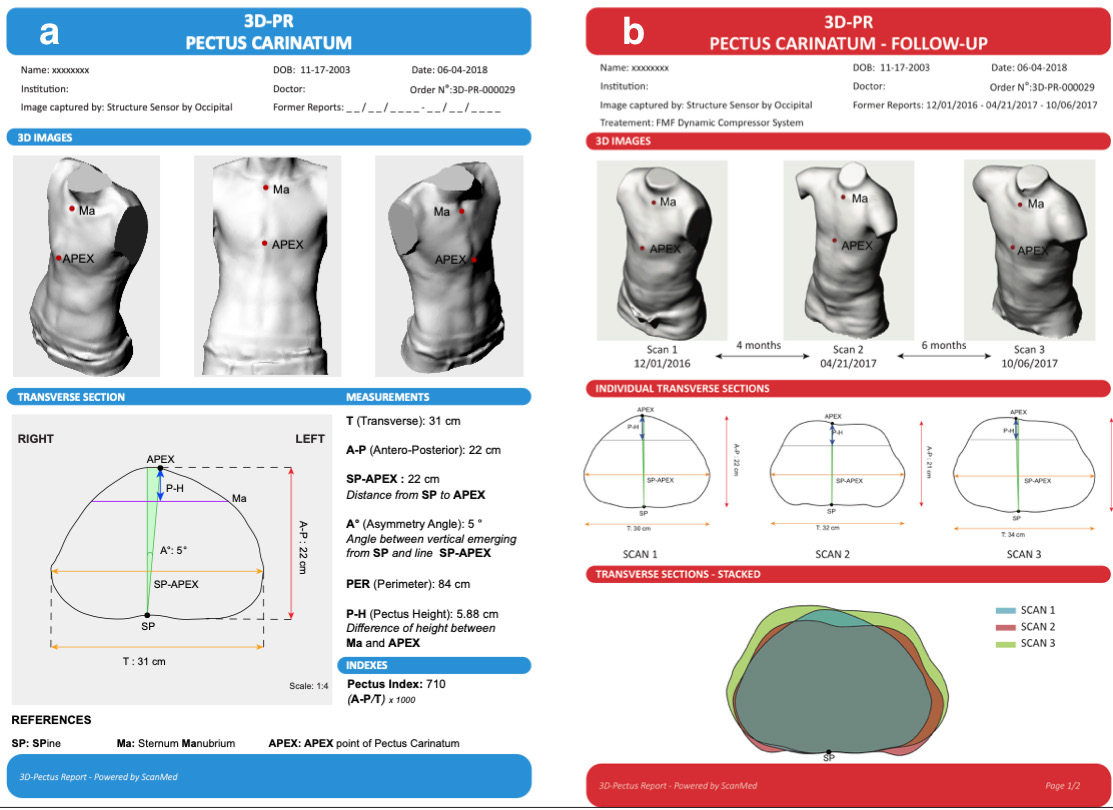
3D scanning technology provides high-quality images that are processed to obtain external chest wall indexes. This resource is particularly useful for (a) the evaluation of severity and (b) reporting comparative results, especially during non-surgical treatments.

Recently, the use of 3D scanners has been reported for the diagnosis, characterisation and follow-up of patients with pectus carinatum and those with excavatum (Fig. 4).^[10,11]^ Three-dimensional virtual reconstruction allows the attending physicians to measure the severity, the symmetry and the evolution of the deformity with treatment, both operative and non-operative, at outpatient clinics. This technology seems promising and avoids radiation, but it still requires validation.

## Treatment

As is the case for other thoracic deformities, treatment in the modern era began in the mid-20th century, with the first report of an attempt at surgical correction by Ravitch in 1952.^[12]^ Since then, surgeons have reported several different variations on the original technique. Following Nuss *et al*.’s^[13]^ report of a new non-resective operation for chest remodelling with a high level of success, many surgeons began exploring and publishing their experiences with non-resective surgeries, which included bar implants for pectus both excavatum and carinatum, both intra- and extrathoracic.

Although dynamic chest compression was described by Haje and Raymundo^[14]^ as early as 1979, this modality did not gain popularity among surgeons until the Nuss technique of chest remodelling (instead of resecting) completely changed the conception of treatment.

This change of paradigm inspired surgeons to explore the concept of reshaping the thorax from the outside, avoiding the need for an operation. In 2008, Martinez-Ferro *et al*. ^[6]^ reported the creation of a pressure-controlled bracing system, and described its application in patients, introducing new concepts such as pressure of initial correction and pressure of treatment, as well as the use of pounds per square inch (psi) as a unit of measure for rigidity of the thoracic cage.

**Fig. 5:**
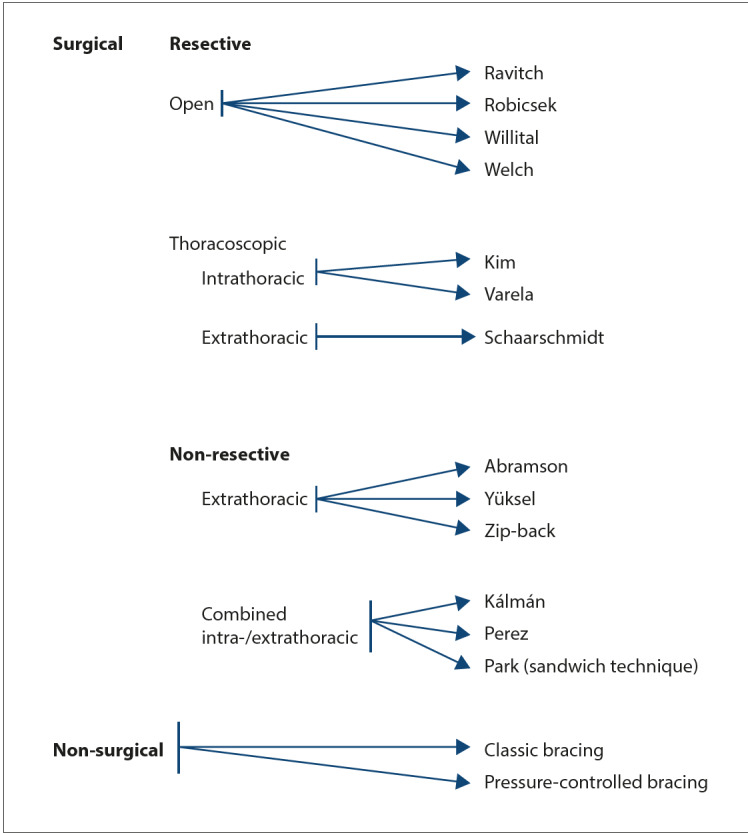
Classification of treatments for pectus carinatum (adapted from Holcomb et al.^[15]^)

In summary, the treatment modalities of pectus carinatum may be either surgical or non-surgical, and surgical treatments are either resective or non-resective (Fig. 5).

### Surgical treatments

Open resective surgeries of the affected cartilages associated with sternal osteotomies were the first operations performed for this thoracic deformity. They consist of large incisions on the chest that are usually submitted to considerable strain, with potential keloid formation, and ample resection of several cartilages, with perichondrium preservation and sternal osteotomies fixed with small bars that require subsequent removal. Disadvantages of this operation include significant blood loss, long operating time and considerable damage to soft tissues.^[16]^ Asphyxiating chondrodystrophy has been reported as a result of open resective surgeries done too early in childhood in pectus excavatum patients (though this is less likely in pectus carinatum cases).^[17,18]^ Far from being abandoned, these surgeries, or variations of them, are still the first choice in cases of very deformed, rigid, non-articulated sternums with associated malformations, or after other approaches have failed. 

### Resective techniques

**Open resective techniques:** The Ravitch,^[12]^ Robicsek,^[19]^ Willital^[21]^ and Welch^[20]^ were the most commonly used open resective techniques prior to Nuss *et al*.’s^[13]^ first report of minimally invasive surgery for pectus excavatum.

**Fig. 6:**
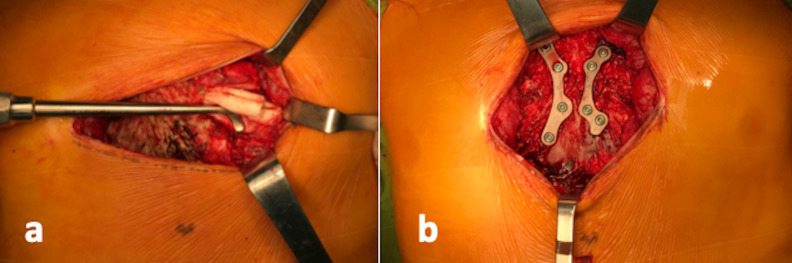
Typical open resective surgery: (a) cartilage resection with preservation of perichondrium, and (b) sternal osteotomy with stabilisation by means of titanium plates and screws.

Ravitch’s^[12]^ first report of an open correction of a pectus carinatum deformity established a series of steps, including cartilage resection of the ribs, sternum osteotomies and fixation. Robicsek *et al*. ^[19]^ reported an upper sternal osteotomy and resection of its lower angulated portion and the xiphoid process. Shamberger and Welch^[20]^ reported on their extensive experience using bilateral cartilage resection, even in cases of pectus carinatum, as well as various osteotomies, depending on the type of sternal deformity. Saxena and Willital^[21]^ reported extensive chondral and distal segments of ribs, detorsion of the sternum with retrosternal mobilisation and fixation with trans-sternal and lateral parasternal metal struts. It is now generally agreed that chondral resection must ensure preservation of the perichondrium to allow for cartilage regrowth (Fig. 6).

**Minimally invasive resective techniques:** Following the introduction of minimally invasive surgery, thoracoscopic options appeared. One of these was the Nuss procedure for pectus excavatum. Thoracoscopic resective correction is further classified as intra- or extrathoracic.

The Kim and Varela techniques are two examples of thoracoscopic resective intrathoracic surgeries for the correction of carinatum deformities. Kim and Idowu^[22]^ reported multiple short chondral and rib segmental resection with preservation of the anterior perichondrium from an intrathoracic aspect under thoracoscopic vision. Varela and Torre^[23]^ extended Kim’s resection, and excised the affected cartilages completely using three ports.

Schaarschmidt *et al*. ^[24]^ reported an extrathoracic resective thoracoscopic approach to pectus carinatum by means of sub-pectoral carbon dioxide dissection. Carbon dioxide insufflation was used to dissect the pectoral muscles from the entire anterior thoracic wall. Through a small incision of 2.9 - 4.7 cm, and the port holes, wedge resections of the cartilages and osteotomies of the sternum were performed as necessary, with subsequent fixation with bars that were removed later.

### Non-resective techniques

Non-resective techniques were the result of the fundamental conceptual change brought about by Nuss *et al*.’s^[13]^ introduction of chest remodelling. The avoidance of resection implies thoracoscopy, with smaller lateral incisions resulting in better cosmesis, less blood loss and shorter operative time. Dystrophy of chondral rib segments is no longer a concern, since growth plates are unaffected. However, these techniques are more effective in flexible thoracic cages that are also amenable to non-operative bracing, so their use in pectus carinatum is gradually decreasing.

Non-resective modalities can be further sub-classified into extrathoracic and combined intra- and extrathoracic.

**Fig. 7:**
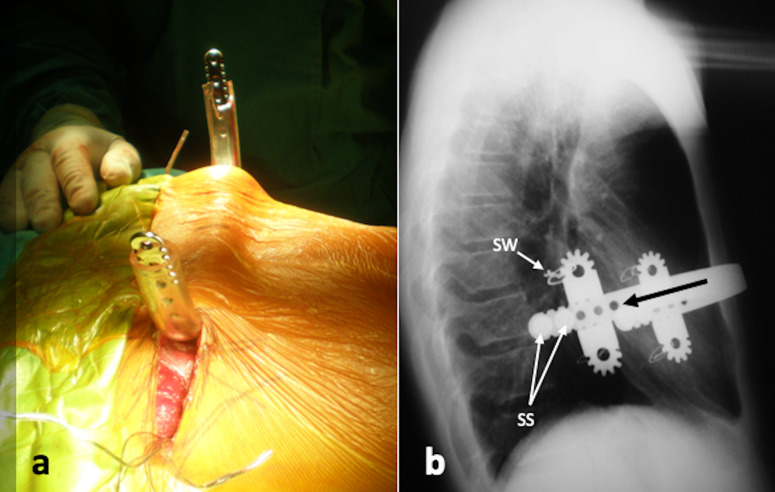
Abramson technique or reverse Nuss, as modified by Martinez-Ferro et al.: (a) a subcutaneous bar is passed pre-sternally; (b) postoperative X-rays showing the bar in place. The stabilisers are fixed to the ribs by means of stainless steel wire (SW). The bar is pushed from front to back (black arrow) using the stabilisers as a guide. Specially designed screws prevent the bar from sliding back (SS).

**Non-resective extrathoracic:** The first report on non-resective extrathoracic treatment of pectus carinatum, or ‘reverse Nuss’, was by Abramson.^[25,26]^ The technique consisted of the placement of a subcutaneous steel bar, introduced through thoracic lateral incisions, and stabilised with two lateral steel devices and steel wire at the incision site (Fig. 7).

**Fig. 8:**
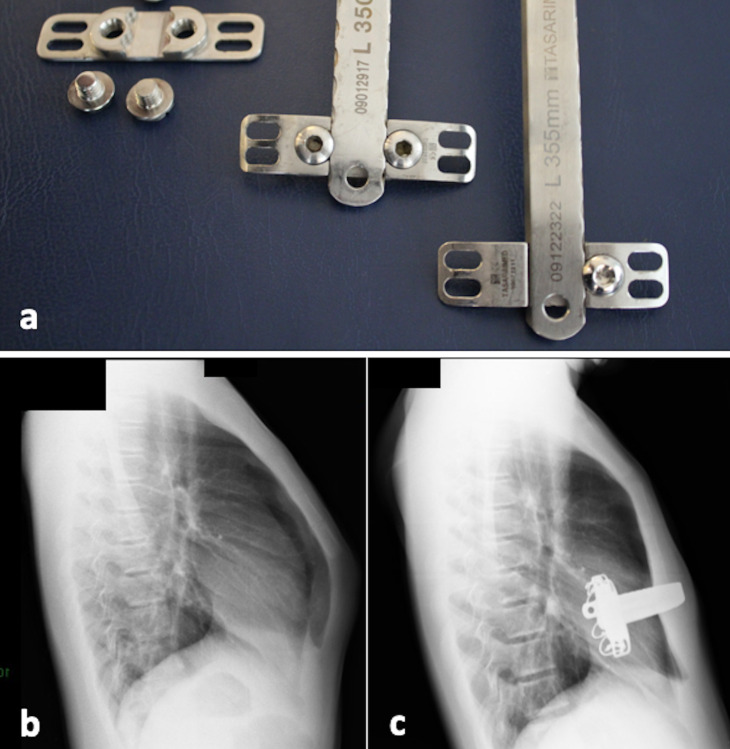
Yüksel’s minimally invasive technique for pectus carinatum: (a) modified extrathoracic implants with different original fixation mechanism; (b) preoperative X-rays; (c) postoperative X-rays.

Modifications of this technique have been published, such as one by Yüksel *et al*. ^[27]^ that uses newly designed implants, the aim of which is to reduce dislocation, one of the most relevant complications of the technique (Fig. 8). In 2018, Yüksel *et al*. ^[28]^ reported his experience with 172 patients over 10 years, and four generations of chest bars, with excellent results in 93.8% of the cases.

**Fig. 9:**
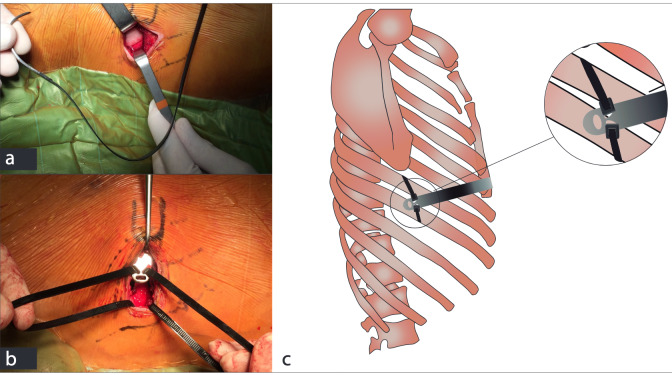
Zip-back technique: the implant is fixed directly to the ribs with specially designed polymer zip-ties. (a) Zip-tie with blunt needle in the tip that will be passed around the dissected rib; (b) zip-ties passed through the ribs, and the implants’ eyelets ready to be fixed; (c) details of the technique.

Recently, Bellia-Munzon *et al*. ^[29]^ have reported the zip-back technique. This approach avoids the use of lateral stabilisers, fixing the implant directly to the ribs by means of specially designed polymer zip-ties (Fig. 9).

**Fig. 10:**
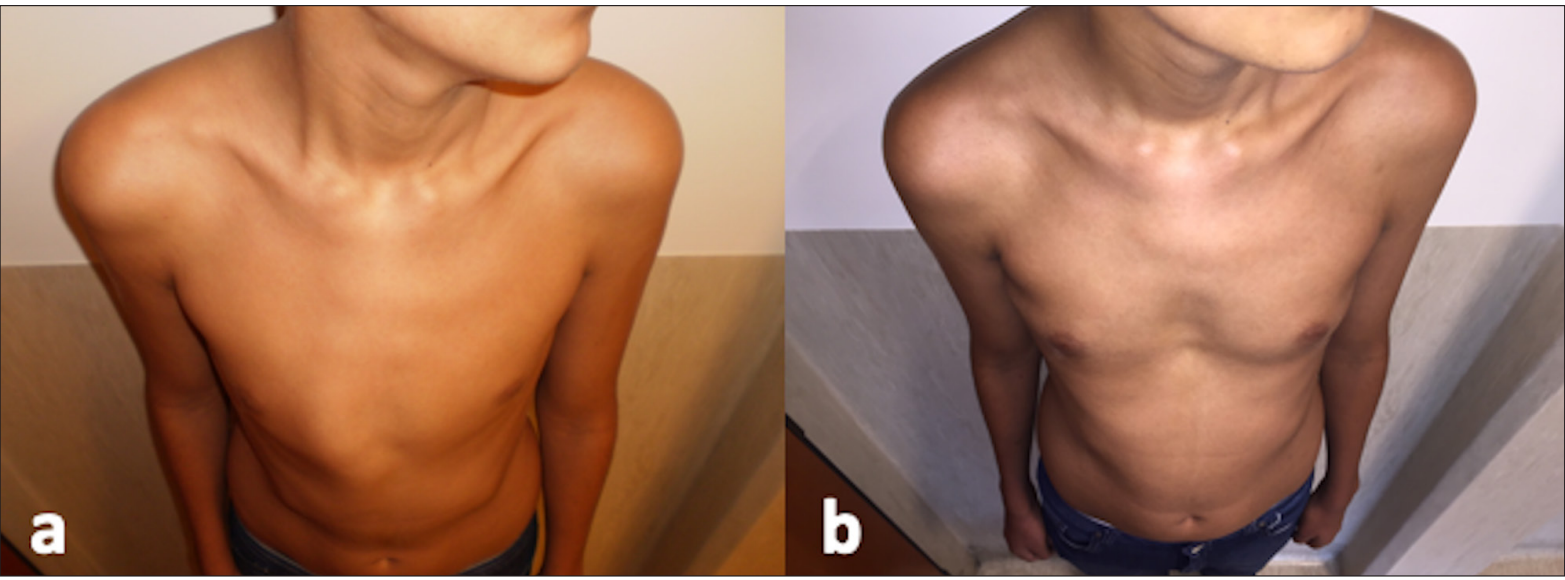
Zip-back technique, before and after: complete reshaping of the thoracic cage is obtained. (a) Preoperative; and (b) 6 months postoperative, showing flattening and widening of the whole anterior chest wall.

In addition to avoiding resection of cartilage and sternal osteotomies, the advantages of non-resective extrathoracic techniques include smaller scars at the side of the chest and a complete reshaping of the chest wall, instead of the local correction of the deformity offered by open resective approaches. This complete remodelling of the chest includes the widening and flattening of the whole anterior chest, with consequent improvement of shoulder and spine posture (Fig. 10).

**Non-resective combined intra-/extrathoracic:** Several authors, such as Kálmán,^[30]^ Pérez *et al*. ^[31]^ and Park and Kim,^[32]^ have reported variations of nonresective combined intra-/extrathoracic surgeries. Kálmán^[30]^ used a modelled bar introduced into the thoracic cavity from the left aspect of the thorax, extruded into the subcutaneous tissue at the point of maximum protrusion of the sternum and reintroduced into the thorax parasternally to the right, fixed with absorbable sutures and meant to avoid the use of stabilisers. Pérez *et al*. ^[31]^ used a steel bar 2 cm longer on each side than the interareolar distance, and introduced it from the right side into the thorax, exited out of the pleural space and advanced it anteriorly to the sternum at the site of maximum protrusion and then reintroduced into the thorax, leaving the tip of the bar inside the pleural space. Finally, Park and Kim^[32]^ developed the ‘sandwich technique’ for asymmetric pectus carinatum or carinatum/excavatum complex. This operation requires two bars, an internal implant for excavatum correction and an external for carinatum, with excellent results.

### Non-surgical treatments

Bracing therapy has gained popularity as a non-operative alternative that has proven to be as good as operative strategies to correct pectus carinatum in patients with flexible thoracic cages and high compliance. This is a good choice as the first line of treatment in almost all cases, and the only solution necessary in many cases with non-complex deformities, flexible thoracic cages and a high level of compliance. This last aspect is the major cause of failure of treatment and abandonment, so a multidisciplinary approach is recommended, with the participation of rehabilitation physicians and physical therapists. There is still a lack of consensus regarding the minimum number of hours patients should wear the brace, and whether bracing may play a role in adult patients. Non-surgical treatments may be classified as classic bracing or pressurecontrolled bracing.

The first reports on bracing for pectus carinatum were published by Jaubert de Beaujeu^[33]^ and Bianchi^[34]^ in the 1960s. Haje *et al*. ^[35–38]^ reported the use of an orthosis referred to as the dynamic chest compressor, and later their extensive experience using the dynamic brace in mild to moderate cases. Since 2000, there have been many reports on the use of classic braces for the treatment of the carinatum deformity.^[40–47]^

**Fig. 11:**
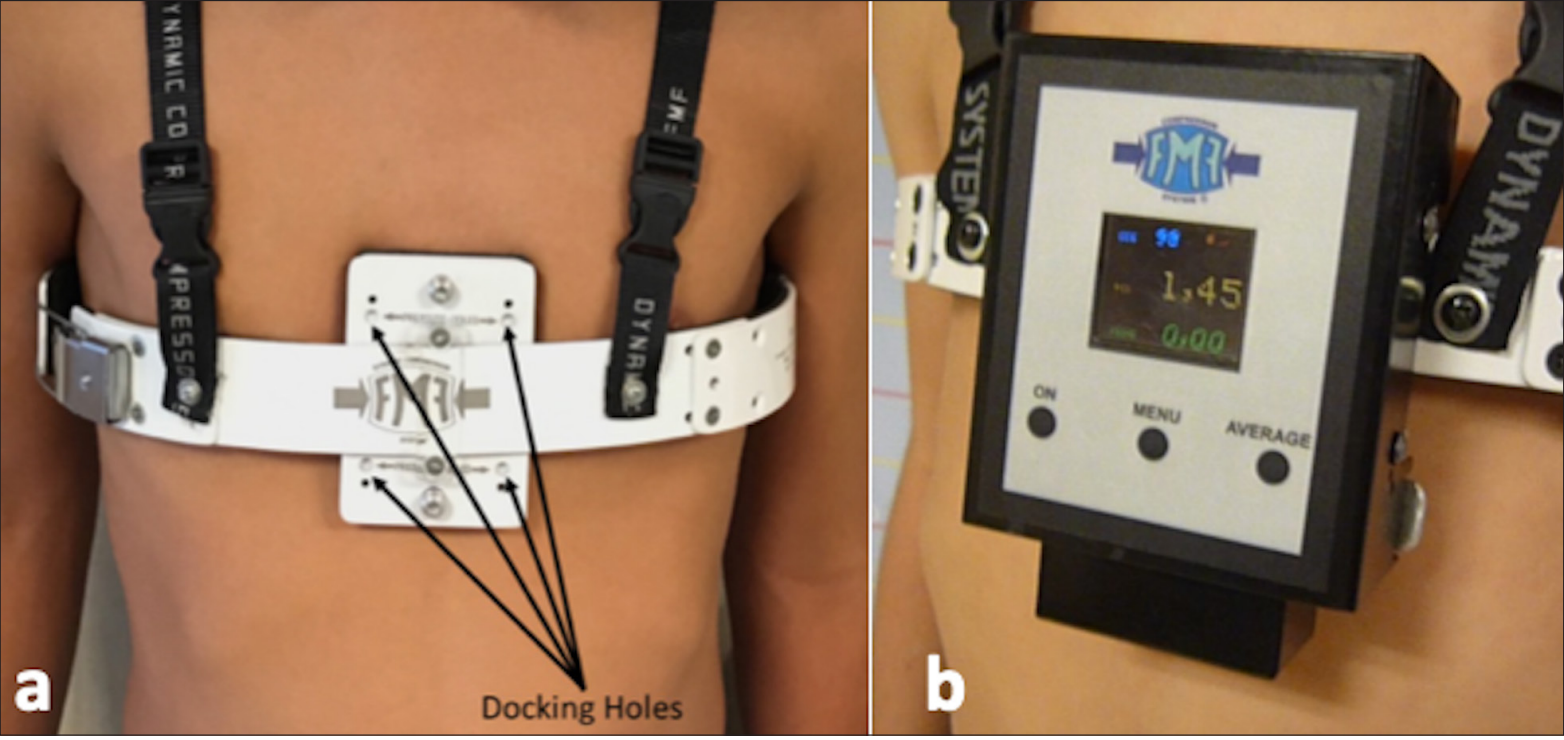
Pressure-measuring devices allow protocolisation of follow-up and determination of cutoff values. (a) FMF Dynamic Compressor System in place. Docking holes permit measuring the pressure of treatment with a device. (b) Pressure-measuring device docked in place.

In 2008, Martinez-Ferro *et al*. ^[6]^ published an 8-year review on the use of a specially designed dynamic compression system (the FMF Dynamic Compression System) (Fig. 11).

This system comprises multiple adjustable elements and a special device that monitors pressure. The concept of pressure of initial correction (PIC) was developed to describe the pressure necessary to completely correct the deformity, by means of measuring the rigidity of the thoracic cage. This permits the doctor to predict prognosis. Pressure of treatment (POT) was also developed to calculate the pressure needed at each adjustment when the patient sees the doctor again. Pounds per square inch (psi) is used as the unit of measure, and pressure under 2.5 psi was considered safe in order to avoid skin lesions. Subsequent authors have also reported their experience with the use of this device: Lopez *et al*. ^[48]^ reported its use in patients with PIC as high as 14 psi, with good results in 61 of 68 cases in 17 months; and Cohee *et al*. ^[49]^ created an algorithm designed to determine the chances of the condition being resolved with a non-operative approach. De Beer *et al*. ^[52]^ validated the use of PIC to predict the duration of treatment, and Poola *et al*. ^[51]^ found that a low PIC at the beginning of treatment was a good prognostic factor. In 2018, de Beer *et al*. ^[50]^ published a systematic review of the measured dynamic compression system. They found 14 studies published between 2008 and 2018, and selected 8 for further analysis. A total of 1 185 patients were included, of whom 44% were still under treatment, 29% had completed treatment successfully, 6% had dropped out and 21% were lost to follow-up. Strategies are needed to decrease rates of abandonment by increasing compliance, which will probably improve results.

**Fig. 12:**
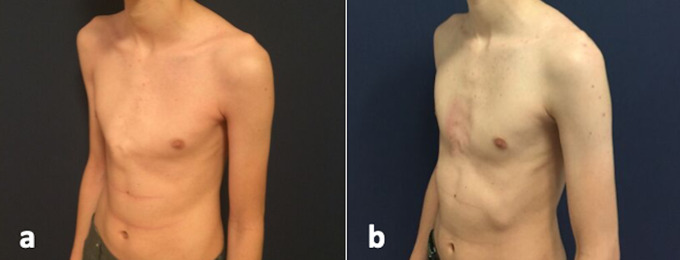
Results obtained with dynamic compression of the chest wall, (a) prior to treatment and (b) after 6 months of treatment.

Overall, the results obtained with bracing are considered better than those obtained with surgical procedures (Fig. 12), making this the first treatment of choice for compliant patients with pectus carinatum.

## Conclusion

We have described the history of the treatment of pectus carinatum, which has changed dramatically over the last 70 years, from the first descriptions of large incisions and wide resections, to minimal access strategies and, finally, to highly efficient non-operative solutions, in a trend towards minimising invasiveness. Today, with a wide variety of choices, treatment modalities are transitioning towards a tailor-made strategy for each patient.
